# Mitophagy: An Emerging Role in Aging and Age-Associated Diseases

**DOI:** 10.3389/fcell.2020.00200

**Published:** 2020-03-26

**Authors:** Guo Chen, Guido Kroemer, Oliver Kepp

**Affiliations:** ^1^The State Key Laboratory of Medicinal Chemical Biology, College of Life Sciences, Nankai University, Tianjin, China; ^2^Gustave Roussy Cancer Campus, Villejuif, France; ^3^INSERM, UMR 1138, Centre de Recherche des Cordeliers, Paris, France; ^4^Equipe 11 Labellisée par la Ligue Nationale Contre le Cancer, Centre de Recherche des Cordeliers, Paris, France; ^5^Université de Paris, Paris, France; ^6^Metabolomics and Cell Biology Platforms, Gustave Roussy Cancer Campus, Villejuif, France; ^7^Sorbonne Université, Paris, France; ^8^Université Paris-Saclay, Faculté de Médecine, Kremlin-Bicêtre, France; ^9^Pôle de Biologie, Hôpital Européen Georges Pompidou, AP-HP, Paris, France; ^10^Suzhou Institute for Systems Medicine, Chinese Academy of Sciences, Suzhou, China; ^11^Karolinska Institute, Department of Women’s and Children’s Health, Karolinska University Hospital, Stockholm, Sweden

**Keywords:** mitophagy, aging, mitochondria, caloric restriction, ROS

## Abstract

Mitochondrial dysfunction constitutes one of the hallmarks of aging and is characterized by irregular mitochondrial morphology, insufficient ATP production, accumulation of mitochondrial DNA (mtDNA) mutations, increased production of mitochondrial reactive oxygen species (ROS) and the consequent oxidative damage to nucleic acids, proteins and lipids. Mitophagy, a mitochondrial quality control mechanism enabling the degradation of damaged and superfluous mitochondria, prevents such detrimental effects and reinstates cellular homeostasis in response to stress. To date, there is increasing evidence that mitophagy is significantly impaired in several human pathologies including aging and age-related diseases such as neurodegenerative disorders, cardiovascular pathologies and cancer. Therapeutic interventions aiming at the induction of mitophagy may have the potency to ameliorate these dysfunctions. In this review, we summarize recent findings on mechanisms controlling mitophagy and its role in aging and the development of human pathologies.

## Introduction

Mitochondria are highly organized and dynamic organelles that undergo continuous fission and fusion (Chen and Chan, 2009; Pham et al., 2012). They originated from endosymbiotic proteobacteria and conferred substantial advantages for eukaryotic cells during evolution. Thus, mitochondria play a critical role in ATP synthesis via oxidative phosphorylation (OXPHOS), β-oxidation regulating fatty acid metabolism, the synthesis of intermediate metabolites through the TCA cycle, as well as calcium homeostasis. On the other hand, like a double-edged sword, mitochondria can turn into a potential threat to cellular homeostasis and survival. In the past decades it has been well documented that mitochondria are the central organelle controlling apoptotic cell death and that the permeabilization of the mitochondrial outer membrane, with the resultant release of pro-apoptotic proteins such as cytochrome c, SMAC/DIABLO, ENDOG, OMI/HTR and AIF, irrevocably leads to cellular demise (Susin et al., 1999; Du et al., 2000; van Loo et al., 2002; Green and Kroemer, 2004; Liu et al., 2009; Wang and Youle, 2009; Li et al., 2017). Moreover, mitochondria are the major source of reactive oxygen species (ROS). During OXPHOS electrons originating mostly from complexes I and III of the electron transport chain, can generate ROS that in turn oxidizes proteins, lipids, and nucleic acids, inside (and outside) the mitochondria, leading to mitochondrial malfunction and cellular damage (Paradies et al., 2000; Hamilton et al., 2001; Short et al., 2005; Miyoshi et al., 2006; Zorov et al., 2014; Redza-Dutordoir and Averill-Bates, 2016). Furthermore, mitochondria serve as an origin of damage associated molecular patterns (DAMP) and in particular mitochondrial DNA (mtDNA), which, once released from mitochondria into the cytosol, can trigger inflammatory responses (Iyer et al., 2009, 2013; Tschopp, 2011; Nakahira et al., 2015; West et al., 2015; Contis et al., 2017).

During aging a wide spectrum of alterations in mitochondrial structure and function can occur. Thus, although cellular antioxidants and free radical scavenging enzymes eliminate most of the generated ROS, a small proportion that escapes clearance can oxidize proteins, lipids and DNA, particularly within the mitochondria. The resulting mutational damage accumulates over lifetime, in particular affecting respiratory chain complexes, which itself results in the overproduction of ROS, forming a vicious cycle that ultimately leads to mitochondrial dysfunction (Greco et al., 2003; Petersen et al., 2003; Short et al., 2005; Lee and Wei, 2012). Morphologically, aging in flies and mammalians manifests with the enlargement of mitochondria, irregular cristae shape and size as well as a decrease in mitochondrial number (Miquel et al., 1980; Terman and Brunk, 2005; Yoon et al., 2006; Leduc-Gaudet et al., 2015). Functionally, OXPHOS activity, and thus ATP synthesis declines with age while ROS production increases in aged animals (Lee and Wei, 2012). As a result, it is not surprising that mtDNA deletions and mutations are detected in tissues from aged animals and humans (Fayet et al., 2002; Eshaghian et al., 2006; Trifunovic, 2006; Lee and Wei, 2012). Consistent with these observations, mtDNA mutator mice that express a proof-reading-deficient version of the mitochondrial DNA polymerase G (POLG) show reduced lifespan and exhibit a premature onset of aging-associated phenotypes including weight loss, reduced subcutaneous fat, alopecia (hair loss), kyphosis (curvature of the spine), osteoporosis, anemia, reduced fertility, and heart enlargement (Trifunovic et al., 2004).

Macroautophagy, which is generally referred to as autophagy, is a conserved intracellular degradation mechanism that removes dangerous, unnecessary or dysfunctional cytoplasmic constituents and invading microbes (Mizushima, 2007; Schuck et al., 2014; Dou et al., 2015; Mochida et al., 2015; Chai et al., 2019). Autophagic activity declines during aging, and autophagy is required for lifespan extension by caloric restriction or caloric restriction mimetics (CRM) such as resveratrol, spermidine, and several chalcones (Eisenberg et al.; Rubinsztein et al., 2011; Lopez-Otin et al., 2016; Madeo et al., 2018; Carmona-Gutierrez et al., 2019). Although the relation between autophagy and aging has been firmly established as an important mitochondrial quality control mechanism, the role of mitophagy in aging and age-related disorders has remained elusive for a long time. However, recent studies have shown that mitophagy has a key function in delaying aging and age-related disorders such as neurodegenerative disorders, cardiovascular pathologies, and cancer. Here, we provide an update on mechanisms that control mitophagy, its role in aging and therapeutic interventions that harness mitophagy to treat age-related disorders.

## Molecular Mechanisms of Mitophagy

**FIGURE 1:**
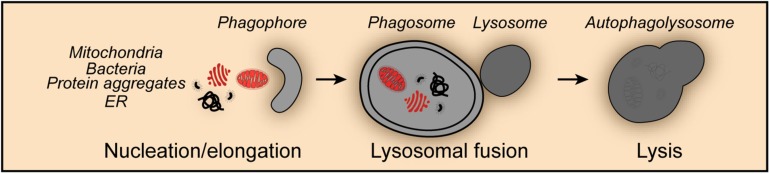
Non-selective mitophagy. Mitophagy shares the core molecular machinery with general macroautophagy and can occur in a non-selective fashion. Thus, mitochondria are engulfed during the nucleation and elongation phase into the forming phagophore together with other cellular content such as protein aggregates, endoplasmic reticulum (ER) derived structures and invasive bacteria. The fusion of the phagosome with lysosomes leads to the formation of the autophagolysosome and the degradation of its content.

Mitophagy shares the core molecular machinery with general macroautophagy and can occur in an either selective or non-selective fashion (Levine and Kroemer, 2019). Thus, during nutrient starvation mitochondria were found in autophagosomes together with cytosolic proteins and organelles such as ER and peroxisomes indicative for non-selective mitophagy (Kopitz et al., 1990; Takeshige et al., 1992; Scott and Klionsky, 1998; Kim et al., 2007; Figure 1). Studies in yeast revealed that mitochondria can be selectively degraded by mitophagy, a process that involves the outer mitochondrial membrane protein SUN family protein Uth1 (Uth1), and type 2C protein phosphatase Ptc6 (Ptc6, better known as Aup1), a phosphatase localizing in the mitochondrial intermembrane space (Petros et al., 1991; Kissova et al., 2004). Mitophagy has been shown to occur under a series of potentially harmful conditions, such as oxidative stress, hypoxia, mitochondrial transmembrane potential loss, the accumulation of unfolded proteins and iron starvation. Moreover, impaired mitophagy and dysfunctional mitophagic mechanisms were associated with numerous physiological and pathological processes including development, differentiation, aging, neurodegenerative disorders, cardiovascular pathologies and cancer.

## PINK1 and Parkin-Regulated Mitophagy

Mutations in PTEN-induced putative kinase 1 (PINK1) and parkin RBR E3 ubiquitin protein ligase (PRKN, better known as Parkin) are associated with autosomal recessive juvenile parkinsonism characterized by motor disturbances and dopaminergic neurodegeneration. Later, genetic analysis showed the molecular mechanism which links PINK1 and Parkin in a common pathway regulating mitophagy, with PINK1 sensing mitochondrial transmembrane potential loss, followed by the recruitment of the E3 ubiquitin ligase Parkin to damaged organelles (Clark et al., 2006; Park et al., 2006). In healthy state, PINK1 led by an N-terminal targeting sequence is imported into mitochondria through the translocase of the outer mitochondrial membrane (TOM) and the translocase of the inner mitochondrial membrane (TIM) complexes, where it is cleaved by matrix processing peptidase (MPP) and presenilins-associated rhomboid-like protein (PARL) (Jin et al., 2010; Deas et al., 2011; Meissner et al., 2011; Greene et al., 2012). Cleaved PINK1 is retro-translocated and released into the cytosol for proteasomal degradation (Yamano and Youle, 2013). However, the loss of mitochondrial transmembrane potential in damaged mitochondria abolishes cleavage, and stabilizes PINK1 on the outer mitochondrial membrane. Recently, the adenine nucleotide translocator (ANT) complex was reported to stabilize PINK1 by inhibiting the pre-sequence translocase TIM23 independently of its nucleotide translocase catalytic activity (Hoshino et al., 2019).

The accumulation of full length PINK1 leads to the phosphorylation (on serine 65) of pre-existing ubiquitin molecules, which are already attached to the outer mitochondrial membrane. Phosphorylated ubiquitin in turn recruits cytosolic Parkin to the mitochondrial membrane and triggers the activation of its ubiquitin ligase activity (Koyano et al., 2014; Wauer et al., 2015). Furthermore, PINK1-dependent phosphorylation of the ubiquitin-like domain of Parkin (Kondapalli et al., 2012; Shiba-Fukushima et al., 2012; Iguchi et al., 2013; Kane et al., 2014) leads to the release of the catalytic RING2 domain and locks Parkin in a functionally active state. Activated Parkin exhibits low substrate specificity and ubiquitylates outer mitochondrial membrane proteins including voltage-dependent anion-selective channel (VDAC) and mitochondrial Rho GTPase (MIRO) proteins (Sarraf et al., 2013; Ordureau et al., 2014; Gladkova et al., 2018).

Studies in cardiomyocytes demonstrated that PINK1 phosphorylates (at serine 442 and threonine 111) mitofusin 2 (MFN2), a GTPase that mediates mitochondrial fusion, which in turn mediates the recruitment of Parkin to damaged mitochondria for mitophagy initiation (Chen and Dorn, 2013; Xiong et al., 2019). Furthermore, it has been suggested that mitochondrial fission might be yet another prerequisite for the initiation of mitophagy. Thus, it was reported that Parkin, among other substrates, ubiquitylates mitofusin 1 (MFN1) and MFN2, leading to their proteasomal degradation, and subsequent mitochondrial fission preceding mitophagy, while the inhibition of mitochondrial fission prevented Parkin-induced mitophagy (Tanaka et al., 2010). Parkin-mediated poly-ubiquitination of outer mitochondrial membrane proteins triggers the recruitment of autophagy receptors such as optineurin (OPTN), calcium binding and coiled-coil domain 2 (CALCOCO2, better known as NDP52) and Tax1 binding protein 1 (TAX1BP1), concomitantly with the activation of the TANK binding kinase 1 (TBK1) that phosphorylates OPTN (at serine 177, 473, and 513) further enhancing its ubiquitin chain binding ability (Wild et al., 2011; Wong and Holzbaur, 2014; Heo et al., 2015; Lazarou et al., 2015). Once recruited to the mitochondria, autophagy receptors can employ initiator proteins from the autophagic machinery such as unc-51 like autophagy activating kinase 1 (ULK1), zinc finger FYVE-type containing 1 (ZFYVE1, better known as DFCP1) and WD repeat domain, phosphoinositide interacting 1 (WIPI1, also known as ATG18) to assemble the autophagosome (Wong and Holzbaur, 2014; Lazarou et al., 2015; Ravenhill et al., 2019; Turco et al., 2019; Vargas et al., 2019) and ATG8s, which could further recruit autophagy receptors to amplify mitophagy signals (Padman et al., 2019). The key function of the ULK1-containing complex for selective autophagy has been recently discussed elsewhere (Turco et al., 2020). Additionally, independently of Parkin, PINK1 may recruit NDP52 and optineurin to mitochondria to directly stimulate mitophagy (Lazarou et al., 2015). It has also been suggested that Parkin mediates the broad proteasomal degradation of outer mitochondrial membrane proteins which leads to membrane rupture and the exposure of the mitophagy receptor prohibitin 2 (PHB2) (Chan et al., 2011; Wei et al., 2017). Conversely, PHB2 can promote PINK1/Parkin-dependent mitophagy by inhibiting the function of PARL and the resultant stabilization of PINK1 on the surface of mitochondria (Yan et al., 2019). However, cells deficient of all Atg8 family members could still undergo mitophagy although the overall size of mitophagosomes is smaller (Nguyen et al., 2016).

Although mutations or deletions of Parkin or PINK1 cause Parkinson disease in humans, mice deficient in either PINK1 or Parkin do not display any related phenotype. However, accumulating evidence shows that Parkinson’s disease is accompanied by immune responses that lead to an increase in serum levels of pro-inflammatory cytokines such as interleukin-6 (IL6), tumor necrosis factor alpha (TNFα), interleukin-1β (IL1B), and interferon gamma (IFNG) (Brodacki et al., 2008; Koziorowski et al., 2012; Lindqvist et al., 2012; Dzamko et al., 2015; Houser and Tansey, 2017; Caggiu et al., 2019). Consistently, the challenge of PINK1 or Parkin deficient mice with immunogenic stress leads to the onset of Parkinson disease-like symptoms (Frank-Cannon et al., 2008; Sliter et al., 2018; Matheoud et al., 2019). Thus, administration of low-dose lipopolysaccharide (LPS) can cause subtle fine-motor deficits and selective loss of dopaminergic neurons in substantia nigra in Parkin deficient mice, although LPS treatment triggered similar persistent neuroinflammation in both wild type and *Parkin^–/–^* mice (Frank-Cannon et al., 2008). The loss of dopaminergic neurons and motoric defects also occur in aged *Parkin^–/–^*; *mutator* mice (Sliter et al., 2018), which accumulate mutations in mtDNA, as well as in *Pink1^–/–^* mice that were orally infected with Gram-negative bacteria (Matheoud et al., 2019). In macrophages, dysfunctional mitochondria are marked by Parkin-dependent ubiquitylation and then recognized by sequestosome 1 (SQSTM1, better known as p62), which is transcriptionally upregulated by nuclear factor kappa B (NF-κB), followed by mitochondrial clearance via mitophagy. This NF-κB and p62-dependent mitophagy pathway prevents excessive inflammation by restraining NLRP3-inflammasome overactivation (Zhong et al., 2016). Moreover, mtDNA released from damaged mitochondria can promote stimulator of interferon response cGAMP interactor 1 (STING1)-dependent interferon regulatory factor 3 (IRF3)-mediated signaling triggering inflammatory response (West et al., 2015), while Parkin-mediated mitophagy prevents inflammation by mitophagic mtDNA clearance (Sliter et al., 2018). Additionally, PINK1 and Parkin signaling can suppress inflammation by repressing mitochondrial antigen presentation delivered by mitochondrial derived vesicles (Matheoud et al., 2016).

The roles of PINK1 and Parkin in heart function have been extensively studied. PINK1 protein levels significantly decrease in humans with end-stage heart failure. PINK1 deficient mice develop left ventricular dysfunction and pathological cardiac hypertrophy, characterized by an increase in oxidative stress and impaired mitochondrial function (Billia et al., 2011). Different from PINK1, Parkin deficiency sensitizes mice to myocardial infarction resulting in reduced overall survival. Morphologically, Parkin deficiency manifests with a disorganized mitochondrial network and a significant decrease in mitochondrial size. Nevertheless, Parkin-deficient mice exhibit normal cardiac function for up to 12 months of age (Kubli et al., 2013). In response to cardiac ischemia, Parkin-mediated mitophagy is induced to mitigate detrimental effects of a prolonged lack of oxygen supply in the heart of wild type mice, indicating the important role of mitophagy for heart homeostasis (Kubli et al., 2013). Simvastatin, an HMG CoA reductase inhibitor used to lower low-density lipoprotein (LDL) and triglycerides levels and thus to prevent heart attack, can stimulate Parkin-dependent mitophagy. Simavastin has the ability to reduce the size of the infarction caused by ischemia/reperfusion in wild-type mice but not in Parkin-deficient animals (Andres et al., 2014). Interestingly, mtDNA released from damaged mitochondria triggers inflammatory responses in cardiomyocytes that culminate in myocarditis and dilated cardiomyopathy (Oka et al., 2012). Moreover, Parkin mediated mitophagy turns over fetal cardiomyocyte mitochondria to facilitate the replacement of mature adult mitochondria, an effect that likely contributes to the perinatal maturation of cardiac metabolism (Kageyama et al., 2014; Gong et al., 2015; Lampert et al., 2019).

## FUNDC1-Mediated Mitophagy

FUN14 domain containing 1 (FUNDC1) is an outer mitochondrial membrane protein with three transmembrane domains, which serves as a mitophagy receptor in mitochondrial uncoupling-, and hypoxia-mediated mitophagy as well as paternal mitochondrial clearance in *C. elegans* (Liu et al., 2012; Chen et al., 2014; Lim et al., 2019). FUNDC1 contains a conserved microtubule associated protein 1 light chain 3 beta (MAP1LC3B better known as LC3)-interacting region (LIR) domain facing the cytosol, which is necessary for its interaction with LC3, a key regulator of autophagy (Liu et al., 2012). FUNDC1-deficiency blocks hypoxia-induced mitophagy, which can be rescued by re-expressing wild-type FUNDC1 but not with a LIR-mutated protein, indicating a key role of LIR-mediated LC3 interaction in FUNDC1 activity (Liu et al., 2012). Indeed, FUNDC1 is constitutively phosphorylated (at tyrosine 18 and serine 13) by the protein kinases SRC proto-oncogene, non-receptor tyrosine kinase (SRC) and casein kinase 2 (CK2), respectively, which reduces its interaction with LC3 (Liu et al., 2012; Chen et al., 2014). Upon hypoxia or loss of mitochondrial transmembrane potential, dephosphorylation (of tyrosine 18 and serine 13) mediated by the mitochondrial phosphatase PGAM family member 5 (PGAM5) and concomitant phosphorylation (of serine 17) by ULK1 enhances the interaction of FUNDC1 with LC3 to promote mitophagy (Liu et al., 2012; Chen et al., 2014; Wu W. et al., 2014). However, the phosphatase responsible for (tyrosine 18) dephosphorylation remains elusive.

The activity of PGAM5 is fine-tuned to regulate FUNDC1-mediated mitophagy, thus during homeostasis PGAM5 activity is inhibited by BCL2-like 1 (BCL2L1 better known as BCL-XL), and the degradation of BCL-XL induced by hypoxia leads to the dephosphorylation of FUNDC1 and the induction of mitophagy (Wu H. et al., 2014). Under oxidative stress conditions, PGAM5 forms multimers to release BCL-XL, which in turn is followed by an increase in BCL-XL phosphorylation and ultimately leads to apoptosis. Once liberated from BCL-XL sequestration, multimeric PGAM5 is able to dephosphorylate FUNDC1, to augment mitochondrial fission and induce mitophagy. Thus, the reciprocal interaction between PGAM5 with BCL-XL and FUNDC1 may serve as a molecular switch between mitophagy and apoptosis under oxidative stress conditions (Ma et al., 2019). Recent studies suggested additional factors such as syntaxin 17 (STX17), a SNARE protein located in the mitochondria-associated membranes (MAM) and mitochondria is also required for PGAM5 to dephosphorylate FUNDC1 during mitophagy (Sugo et al., 2018).

Moreover, in addition to this tight control, mitochondrial dynamics participate in FUNDC1-mediated mitophagy. Thus, it was reported that FUNDC1 interacts with both the mitochondrial fission key factor dynamin 1 like (DNM1L, better known as DRP1) and inner membrane fusion regulator OPA1 mitochondrial dynamin like GTPase (OPA1) to coordinate mitochondrial dynamics and mitophagy. Mitophagic stress stimulates the disassembly of the FUNDC1-OPA1 complex, while enhancing the association of FUNDC1 with DRP1, leading to mitochondrial fission, thus fostering mitophagy (Chen et al., 2016). FUNDC1 was described to associate with the ER protein calnexin (CANX) in mitochondria-associated ER membranes (MAMs). During hypoxia, the association between FUNDC1 and CANX is decreased, thereby liberating FUNDC1 for its interaction with DRP1, triggering mitochondrial fission and mitophagy (Wu W. et al., 2016). Interestingly, membrane associated ring-CH-type finger 5 (MARCHF5) can ubiquitylate FUNDC1 for proteasomal degradation, and desensitize mitochondria to hypoxia-induced mitophagy thus constituting a negative regulation mechanism at early stages of hypoxia (Chen et al., 2017).

The physiological role of FUNDC1 has been studied in detail and it has been shown that FUNDC1 plays an important role in liver cancer and obesity. In a mouse model of human hepatocellular carcinoma (HCC) induced by the chemical carcinogen, diethylnitrosamine (DEN), liver specific knockout of FUNDC1 facilitates the cytosolic release of mtDNA due to a defect in mitophagy, resulting in an accumulation of dysfunctional mitochondria, an elevated release of proinflammatory cytokines, such as IL1B and hyperproliferation of hepatocytes, finally culminating in the initiation and progression of DEN-induced HCC (Li et al., 2019). Furthermore, skeletal-muscle-specific knockout of FUNDC1 impairs mitochondrial energetics and negatively affects physical fitness. However, FUNDC1 deficiency decreases the susceptibility to high-fat-diet-induced obesity with improved insulin sensitivity and glucose tolerance. In fact, FUNDC1 deficiency elicits a retrograde response in muscle with an upregulation of fibroblast growth factor 21 (FGF21) expression, and thereby promotes the thermogenic remodeling of adipose tissue (Fu et al., 2018). FUNDC1 and BCL2 interacting protein 3 like (BNIP3L, better known as NIX) but not PINK1/Parkin-dependent mitophagy facilitates the removal of impaired mitochondria and thus maintains mitochondrial network reorganization during cardiac progenitor cell (CPC) differentiation. Interestingly, mice expressing a proofreading-defective mitochondrial DNA polymerase G gamma (*PolG^*D*257*A/D*257*A*^*), experience premature aging and develop accelerated age-related cardiomyopathy due to the accumulation of mtDNA mutations (Lampert et al., 2019).

## BNIP3 and NIX-Dependent Mitophagy

BCL2 interacting protein 3 (BNIP3) and NIX, belong to the BH3 only domain proteins of the BCL2 family, which localize at the outer mitochondrial membrane and are involved in stress sensing and the induction of cell death when cellular stress prevails (Zhang and Ney, 2009). More recently, the role of BNIP3 and NIX in autophagy has been extensively studied. Both BNIP3 and NIX are hypoxia-inducible genes (Bruick, 2000; Sowter et al., 2001; Kubasiak et al., 2002), and play an important role in hypoxia-induced macroautophagy and mitophagy (Zhang and Ney, 2009). An increase in BNIP3 protein levels can lead to the liberation of Beclin1 (BECN1) from BCL2 apoptosis regulator (BCL2) and/or BCL-XL sequestration to initiate mitophagy, to prevent ROS production and subsequent cell death (Zhang et al., 2008).

NIX is known for its prominent function in the mitophagy-dependent maturation of red blood cells. Mammalian erythroid cells undergo enucleation and the removal of organelles during terminal differentiation, in which the maturation process of enucleated immature reticulocytes to erythrocyte necessitates complete mitochondrial clearance depending on NIX (Schweers et al., 2007; Sandoval et al., 2008). During erythrocyte differentiation NIX expression is significantly increased, and leads to a decrease in mitochondrial transmembrane potential and the induction of mitophagy (Aerbajinai et al., 2003). Cells from Nix-deficient mice exhibit defects in the incorporation of mitochondria into autophagosomes and further autophagosomal maturation (Schweers et al., 2007; Sandoval et al., 2008). Furthermore, the elimination of mitochondria does not require the core autophagic gene ATG5, but depends on the autophagic kinase ULK1, indicating a specific function of ULK1 in mitophagy during red blood cell maturation (Kundu et al., 2008; Honda et al., 2014).

Mechanistic analysis indicated that NIX functions as a mitophagy receptor that interacts with LC3 via its LIR domain and thus recruits LC3 family proteins to damaged mitochondria. Ablation of the NIX-LC3/GABA type A receptor-associated protein (GABARAP) interaction retards mitochondrial clearance in maturing murine reticulocytes (Novak et al., 2010). Similarly, the mutation of the LIR motif within the BNIP3 gene leads to the ablation of BNIP3-LC3 interaction and impairs mitophagy and ERphagy, although it does not affect the pro-death activity of BNIP3 (Hanna et al., 2012). Interestingly, the interaction of BNIP3 and NIX with LC3 are fine-tuned by the phosphorylation state of moieties adjacent to the LIR domain. Thus, the phosphorylation of serine 17 and serine 24 flanking the BNIP3 LIR motif promotes its binding affinity to LC3 and GABA type A receptor associated protein like 2 (GABARAPL2) (Zhu et al., 2013). Likewise, phosphorylation of NIX (at serine 34 and 35) in close proximity to the LIR stabilizes the NIX-LC3 complex and enhances autophagosomal recruitment to mitochondria (Rogov et al., 2017). However, the kinases and phosphatases specific for BNIP3 and NIX phosphorylation remain elusive. Moreover, high oxidative phosphorylation activity leads to the recruitment of the small GTPase Ras homolog, mTORC1 binding (RHEB) to the mitochondrial outer membrane which promotes mitophagy through physical interaction with NIX and LC3 (Melser et al., 2013).

In several human cancer types, including hematological malignancies, lung, breast, gastric, pancreatic, and liver cancer, the epigenetic silencing of BNIP3 expression is reported to correlate with invasiveness and metastasis (Okami et al., 2004; Koop et al., 2009; Chourasia et al., 2015). Conversely, some studies indicate that BNIP3 and NIX are upregulated in human breast ductal carcinoma *in situ*, which manifest with high-grade, necrotic lesions and invasive tumors (Sowter et al., 2001, 2003). In malignant glioma cells, ceramide induces autophagic cell death via lethal mitophagy (Sentelle et al., 2012), through the activation of BNIP3 (Daido et al., 2004). However, the role of BNIP3- and NIX-mediated mitophagy in cancer has to be carefully reevaluated, considering the fact that BNIP3 and NIX are proapoptotic BH3-only proteins. Furthermore, the expression of BNIP3 is upregulated in post-natal ventricular myocytes and adult rat hearts subjected to hypoxia, and in animals that exhibit a chronic heart failure, which is associated with myocardial cell death. Both the pan-caspase inhibitor z-VAD-fmk and the mitochondrial permeability transition pore (MPTP) inhibitor bongkrekic acid prevent BNIP3-induced mitochondrial defects and cell death (Regula et al., 2002). In yet another ischemia model, hypoxia upregulates mRNA and protein levels of BNIP3, while acidosis stabilizes the protein and increases its association with mitochondria for the induction of cell death (Kubasiak et al., 2002). In ischemia induced injury, BNIP3 is engaged in pro-death signaling, whereas its role in mitophagy in this setting needs further investigation (Hamacher-Brady et al., 2007). It has been reported that mitophagy is significantly impaired in neurodegenerative disorders such as Alzheimer’s disease (AD) and Ataxia telangiectasia (A-T), while mitophagy stimulation induces beneficial effect including an increase in cognition and an extended lifespan in a NIX- or PINK1 and Parkin-dependent manner (Fang et al., 2016, 2019). Mitophagy restoration enhances the phagocytic efficacy of microglia to diminish the aggregation of insoluble amyloid-β, and thus reduces pro-inflammatory factors such as IL6 and TNFA while increasing the anti-inflammatory cytokine interleukin-10 (IL10) which has been shown to promote mitophagy in macrophages (Ip et al., 2017; Fang et al., 2019).

## ATG32/BCL2L13-Controlled Mitophagy

In yeast, mitophagy selectively occurs in post-log phase cells under respiratory conditions. Mitophagy protein Atg32 (Atg32) is a transmembrane protein imbedded in the outer mitochondrial membrane with a ubiquitin-like protein Atg8 (Atg8) interacting-motif (AIM) for the recruitment of autophagosomes (Okamoto et al., 2009). In addition, Atg32 interacts with the adaptor autophagy protein Atg11 (Atg11) to facilitate the incorporation of mitochondria into the nascent autophagic vacuole (Kanki et al., 2009). The activity of Atg32 is additionally regulated via proteolytical cleavage by the mitochondrial i-AAA protease Yme1 (Yme1), which is essential for the interaction between Atg32 and Atg11 and the induction of mitophagy (Wang et al., 2013). Atg32 activity is further fine-tuned via the phosphorylation at Ser114 and Ser119 by casein kinase 2 (CK2) downstream of the mitogen-activated protein kinases (MAPK) Hog1 and Pbs2 to promote its interaction with Atg11 (Aoki et al., 2011; Mao et al., 2011; Kanki et al., 2013). Alternatively, yet another MAPK signaling pathway implicating Slt2 can regulate both mitophagy and the selective degradation of peroxisomes (pexophagy), although the mechanism remains elusive (Mao et al., 2011). Mitochondrial dynamics appear to constitute another regulatory instance for the induction of mitophagy in yeast. Thus, Atg11 recruits the fission machinery to mitochondria via its interaction with Dnm1 to segregate degrading mitochondria from the network for mitophagy (Mao et al., 2013). The mammalian homolog of Atg32 has been identified as Bcl-2-like protein 13 (BCL2L13), which also contains a LIR domain to interact with LC3 and can induce mitophagy in mammalian cells and Atg32 deficient yeast (Murakawa et al., 2015, 2019). A recent study indicated that Atg32 might be implicated in age asymmetry between the mother and daughter cells in yeast (Jiang et al., 2019). However, the detailed roles of Atg32 and/or BCL2L13 in aging and age-related diseases need further research.

## Other Mitophagy Receptors

During recent years with increasing interest in the exploration of mitophagy, additional mitophagy receptors have been identified to mediate mitophagy including autophagy and beclin 1 regulator 1 (AMBRA1), which acts in a PARKIN- and p62-independent manner (Di Rita et al., 2018; Strappazzon et al., 2019), FK506 binding protein 8 (FKBP8) that specifically interacts with microtubule associated protein 1 light chain 3 alpha (MAP1LC3A better known as LC3A) and thus facilitates mitophagy (Bhujabal et al., 2017), and NLR family member X1 (NLRX1) which contains an LIR domain and is harnessed by Listeria during infection to induce mitophagy for its survival in macrophages (Zhang et al., 2019). Interestingly, upon mitochondrial depolarization, 4-nitrophenylphosphatase domain and non-neuronal SNAP25-like protein homolog 1 (NIPSNAP1) and NIPSNAP2 translocate from the mitochondrial matrix to the surface of the organelle and recruit autophagy receptors and ATG8 proteins for mitophagy. It is worthy to note that NIPSNAP1-deficient zebrafish larvae display parkinsonian phenotypes, including the loss of tyrosine hydroxylase (Th1)-positive dopaminergic (DA) neurons, reduced motor activity, and increased oxidative stress, as well as reduced mitophagy in the brain (Princely Abudu et al., 2019).

Lipids can also function as mitophagy receptors by interacting with LC3. Thus, ceramide has been reported to target autophagolysosomes to mitochondrial membranes and provoke lethal mitophagy (Sentelle et al., 2012). However, in acute myeloid leukemia (AML) cells, ceramide synthesis is suppressed by Fms-like tyrosine kinase 3 (FLT3)-internal tandem duplication (ITD) signaling, which confers its resistance to cell death. Molecular or pharmacologic inhibition of FLT3-ITD in AML cells reactivated ceramide synthesis, mitochondrial division, mitophagy and cell death, indicating a potential application for the therapeutic induction of mitophagy in cancer (Dany et al., 2016). While cardiolipin, a phospholipid mainly localized at the inner mitochondrial membrane, can externalize to the outer membrane and serve as a mitophagy receptor in neuronal cells (Chu et al., 2013). Cardiolipin mediated mitophagy has been shown to play an important role in traumatic brain injury (TBI) by removing damaged mitochondria thus mitigating ROS overproduction and decreasing apoptosis (Chao et al., 2019).

## Piecemeal Mitophagy

**FIGURE 2:**
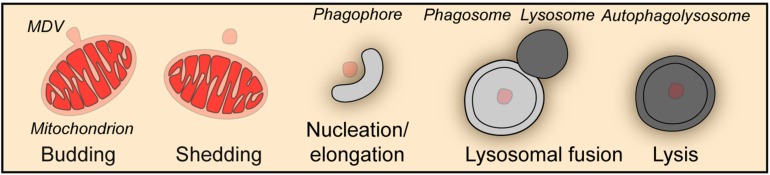
Piecemeal mitophagy. Mitophagy can occur through the formation of mitochondria-derived vesicles (MDV), which in turn are degraded by the autophagic machinery in a piecemeal fashion.

Besides the wholesale mitophagy described above, a piecemeal mitophagy mechanism exists to deliver small vesicles budded off from mitochondria to lysosomes for degradation, which is important for the maintenance of mitochondrial homeostasis (Figure 2). In a screen aiming at the identification of autophagic protein substrates, metaxin1 (MTX1) was shown to be degraded by piecemeal mitophagy, in which MTX1-containing vesicles are segregated from mitochondria and then degraded by lysosomes in a microtubule associated protein 1 light chain 3 gamma (MAP1LC3C better known as LC3C)- and p62-dependent manner (Le Guerroue et al., 2017). When mitochondria face unfolded protein stress, PINK1 and Parkin facilitate a DRP1-dependent segregation of mitochondrial subdomains from the network for degradation by mitophagy to prevent proteotoxicity spreading (Burman et al., 2017). Furthermore, under oxidative stress, TOMM20 positive mitochondrial derived vesicles deliver oxidized proteins to lysosomes for degradation (Soubannier et al., 2012a, b). Strikingly, this process does not require ATG5 or LC3, but is driven by PINK1 and Parkin and depends on syntaxin 17 (STX17) to mediate the fusion between vesicles and endolysosomes (Soubannier et al., 2012a; McLelland et al., 2014, 2016).

## The Role of Mitophagy in Aging

Heteroplasmy of mtDNA is a hallmark of aging. The homogeneity of mtDNA in newborn life is ensured by the selective removal mechanism of deleterious mtDNA in the female germline (Lieber et al., 2019) and paternal mitochondrial removal after fertilization (Al Rawi et al., 2011; Sato and Sato, 2011; Politi et al., 2014; Rojansky et al., 2016; Sato et al., 2018), in both of which mitophagy is highly involved. As mtDNA mutations and deletions accumulate with age, which are associated with a variety of diseases, such as cancer, neurodegenerations, and cardiovascular diseases (Liu et al., 1996; Petros et al., 2005; Sharma et al., 2005; Wallace, 2005; Turnbull et al., 2010), mitochondrial respiration activity and mitochondrial function are damaged, which lead to decreased mitochondrial potential. It has been reported that Parkin is recruited to mitochondria with low potential and required for the mitophagic degradation of malfunctional mitochondria with mtDNA mutations (Gilkerson et al., 2012). And long-term overexpression of Parkin can increase the ratio between the mitochondria with wild type mtDNA and the ones with deleterious COXI mutations (Suen et al., 2010). Interestingly, in mice, even heteroplasmy of normal mtDNA leads to reduced activity, food intake, respiratory exchange ratio; accentuated stress response; and cognitive impairment (Sharpley et al., 2012), which might be related to the absence of mitophagy-dependent elimination of paternal mitochondria. Although mitochondria are mostly of maternal origin, resulting from the mitophagy-dependent clearance of paternal mitochondria, exceptional cases are reported in human (Luo et al., 2018), sheep (Zhao et al., 2001), mouse (Gyllensten et al., 1991), and drosophila (Nunes et al., 2013; Dokianakis and Ladoukakis, 2014) in which paternal inheritance of mtDNA and thus mtDNA heteroplasmy exist. In *C. elegans*, mitophagy-dependent paternal mitochondrial elimination has been extensively studied, and delayed clearance of paternal mitochondrial after fertilization leads to an increase in embryonic lethality (Zhou et al., 2016). However, the effect of normal mtDNA heteroplasmy on aging needs further research.

The involvement of mitophagy in aging has been extensively studied in *C. elegans*. Mitophagy mediated by dct-1, the ortholog of NIX, plays an important role during *C. elegans* aging. Mitochondria accumulate with age in wild type worms, and deficiency in *dct-1*, as well as the autophagy key gene bec-1, recapitulates the effect of aging on mitochondrial mass in young adult animals. Pronounced induction of mitophagy was observed in long-lived *daf-2* mutants, and impairment of mitophagy by knockdown of *dct-1*, *pink-1*, and *pdr-1* (the nematode Parkin homolog) significantly shortens the lifespan of *daf-2* mutants. In fact, *dct-1* is transcriptionally induced under the control of *skn-1* and *daf-16* [the nematode homolog of mammalian nuclear factor, erythroid 2 like 2 (NFE2L2, better known as NRF2) and forkhead box O3 (FOXO3), respectively] to remove dysfunctional mitochondria via mitophagy and coordinate mitochondrial biogenesis and mitophagy (Palikaras et al., 2015). Mitochondrial biogenesis and mitophagy may cooperate to antagonize the aging process (Palikaras et al., 2015; Fang et al., 2017). Interestingly, tomatidine, a natural compound abundant in unripe tomatoes, inhibits age-related skeletal muscle atrophy in mice and extends health- and life-span in *C. elegans*. Mechanistic analysis showed that tomatidine stimulates mitochondrial biogenesis and PINK1- and DCT1-related mitophagy and increases healthspan (Fang et al., 2017). Moreover, *dct-1*, *pink-1*, and *pdr-1* are engaged in lifespan extension induced by mild mitochondrial stress achieved by frataxin depletion-induced iron-starvation in *C. elegans* (Schiavi et al., 2015). Excessive iron chelation also stimulates mitophagy in mammalian cells, which however does not require PINK1 or Parkin activation but depends on glycolysis (Allen et al., 2013).

Exercise has long been known to promote healthy aging and decrease the susceptibility to age-related diseases probably, depending on the induction of autophagy (He et al., 2012; Escobar et al., 2019). Mitophagy may also be involved in the beneficial effects of exercise. A recent study has shown that exercise activates the AMPK-ULK1 cascade to provoke the removal of damaged mitochondria via mitophagy. Moreover, exercise improves glucose tolerance in wild type mice but not in ULK1 deficient mice (Laker et al., 2017).

Caloric restriction is yet another way to extend healthy lifespan. Similar to exercise, nutrient deprivation activates the AMPK-ULK1 cascade that is required for mitophagy to remove damaged mitochondria and promote cellular survival (Egan et al., 2011). Nutrient starvation causes the rapid depletion of cytosolic acetyl-coenzyme A, and subsequently reduces the activity of the acetyltransferase E300, which is known to acetylate ATG proteins and to inhibit their pro-autophagic function (Lee and Finkel, 2009; Marino et al., 2014). The depletion of general control of amino acid synthesis 5-like 1 (GCN5L1), a component of the mitochondrial acetyltransferase machinery that counteracts deacetylation mediated by SIRT3 (Scott et al., 2012), results in p62 and Atg5-mediated mitochondrial autophagy (Webster et al., 2013). Furthermore, the depletion of GCN5L activates both the transcription factor EB (TFEB), which is a master regulator of autophagy, and PPARγ coactivator 1α (PGC-1α), which controls mitochondrial biogenesis, coordinating the turnover and biogenesis of mitochondria (Scott et al., 2014).

Due to the difficulties to maintain long-term caloric restriction, the concept of caloric restriction mimicry has been developed (Madeo et al., 2019). The intracellular concentration of spermidine, a natural polyamine and prototype caloric restriction mimetic (CRM), declines during aging, and the administration of spermidine can extend the lifespan of yeast, flies and worms, and human immune cells (Eisenberg et al., 2009). Interestingly, spermidine stimulates mitophagy in cardiomyocytes of both young and aged mice, which might impinge on spermidine-mediated cardioprotection (Eisenberg et al., 2016). However, the role of mitophagy in spermidine induced lifespan extension needs further investigation. Aspirin, another CRM, induces autophagy by inhibiting EP300 and stimulates mitophagy in the heart of mice (Pietrocola et al., 2018). Additionally, *dct-1*, the *C. elegans* ortholog of the mammalian mitophagy receptor NIX and BNIP3, mediates longevity and mitophagy in nematodes (Palikaras et al., 2015), and silencing of *dct-1* abolished aspirin induced autophagy in *C. elegans* (Pietrocola et al., 2018). Different from spermidine and aspirin which stimulate autophagy by inhibiting acetylase EP300, induction of autophagy by resveratrol, a naturally occurring polyphenol (and yet another CRM), requires the nicotinamide adenine dinucleotide–dependent deacetylase sirtuin 1 (SIRT1) (Morselli et al., 2011). Apparently, resveratrol has the capacity to induce mitophagy through increasing the expression of PINK1, Parkin, and Beclin1, and AMPK activation by resveratrol participates in neurodegenerative diseases, cerebral ischemia, muscular dystrophy, and inflammation (Ferretta et al., 2014; Wu J. et al., 2016; Sebori et al., 2018; Wang et al., 2018; Cao et al., 2019; Pineda-Ramirez et al., 2019).

Additional compounds exert their lifespan extending effect via mitophagy. Thus, urolithin A, the end-products of both ellagitannins and ellagic acid, extends lifespan and improves fitness during *C. elegans* aging and improves muscle function and exercise capacity in rodents. In-depth analysis demonstrates that mitophagy is required for the beneficial effect of urolithin A (Ryu et al., 2016). Recently, it was reported that Urolithin A reverses memory impairment through PINK1-, PDR1-, or DCT1-dependent mitophagy in both amyloid-β (Aβ) and tau *C. elegans* models of Alzheimer’s disease (Fang et al., 2019). A clinical investigation suggests that urolithin A improves mitochondrial and cellular health following regular oral consumption in humans (Andreux et al., 2019). However, one report suggests that urolithin A stimulates autophagy but not mitophagy to inhibit ER stress in a model of ischemic neuronal injury (Ahsan et al., 2019).

Nicotinamide adenine dinucleotide (NAD) is a critical metabolite involved in many physiological processes, including metabolism, post-translational protein modification, and DNA repair and its concentration is closely associated with aging. NAD levels decrease with age, while the upregulation or replenishment of NAD metabolism has been shown to exhibit beneficial effects against aging and age-associated diseases (Li et al., 2001; Mouchiroud et al., 2013; Yaku et al., 2018). Treatments that increase intracellular NAD^+^ improve mitochondrial quality via mitophagy and thus extend health- and life-span in Ataxia Telangiectasia models and reverse cognitive deficits in models of Alzheimer’s disease (Fang et al., 2016, 2019). Sirtuins, whose activity depend on NAD^+^, may also participate in NAD^+^ administration stimulated mitophagy, and it appear that their function declines with aging (Li et al., 2001; Mouchiroud et al., 2013; Feldman et al., 2015; Kerr et al., 2017). Interestingly, in response to oxidative stress, SIRT3, a mitochondrial sirtuin, deacetylates the transcription factor FOXO3 to regulate BNIP3, NIX and LC3 expression, thereby stimulating mitophagy as well as mitochondrial biogenesis and dynamics (Tseng et al., 2013).

Rapamycin, an allosteric inhibitor of mechanistic target of rapamycin (mTOR), prolongs life in yeast, worms, flies, and mice. Rapamycin also prevents age-related conditions in rodents, dogs, nonhuman primates, and humans (Blagosklonny, 2019). mTOR is a critical nutrient sensor and has multiple downstream effects, including protein synthesis, and autophagy. Recent studies indicate that eliminating damaged mitochondria via mitophagy may be one of the mechanisms responsible for the beneficial effects of rapamycin. Tuberous sclerosis complex 2 (TSC2) is upstream of mTOR and its inhibition leads to constitutive mTOR activation. Interestingly, TSC2 deficiency impairs mitophagic flux, as indicated by reduced expression of PINK1 and PARK2 translocation to uncoupled mitochondria, a defect that can be restored with rapamycin administration (Bartolome et al., 2017). Moreover, axonal and global mitophagy of damaged mitochondria is impaired in neuronal *in vitro* and *in vivo* models of tuberous sclerosis complex, contrasting with the fact that blocking mTORC1 or inducing mTOR-independent autophagy restores mitochondrial homeostasis (Ebrahimi-Fakhari et al., 2016). In another study, rapamycin significantly enhanced mitophagy by increasing the translocation of p62 and Parkin to the damaged mitochondria in a mouse spinal cord injury model (Li et al., 2018). Consistent with these findings, PINK1 and Parkin-dependent mitophagy is impaired and mTOR is hyperactivated in primary human fibroblasts derived from individuals with Down syndrome. In this context, inhibition of mTOR using AZD8055 restores autophagic flux, as well as mitophagy initiated by PINK1 and Parkin (Bordi et al., 2019).

## Perspectives

**FIGURE 3:**
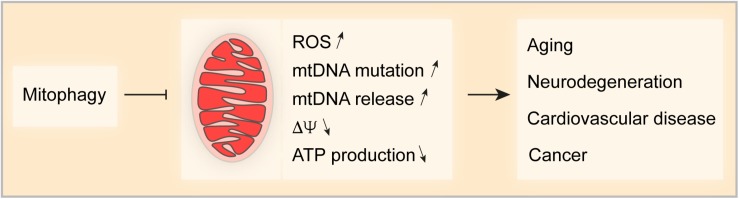
Impact of Mitophagy on age-related pathologies. Mitophagy is a key mechanism for mitochondrial quality and quantity control. Thus, mitophagy limits the production of reactive oxygen species (ROS), the accumulation of mutations in and the release of mitochondrial DNA (mtDNA), appearance of transmembrane potential loss and the decrease in ATP production. Taken together, mitophagy controls various factors that can drive pathologies such as aging-related disorders and neurodegeneration, cardiovascular disease and cancer.

Mitochondria are important for cellular life and death, implying that mitochondrial homeostasis must be tightly controlled and fine-tuned when cells respond to stress. Mitophagy is the primordial mechanisms for mitochondrial quality and quantity control and multiple mechanisms control this process. Some studies indicate an ample crosstalk between different mitophagy pathways that may coordinate and complement to deal with environmental challenges. Nevertheless, the detailed mechanism that link the different pathways in the complex network of mitophagy control need further investigation (Chen et al., 2014; Gao et al., 2015; Zhang et al., 2016). Dysfunction of mitochondria is one of the major characteristics of aging and age-related disease. Increasing evidence shows that mitophagy (by removing damaged mitochondria) is significantly involved in counterbalancing age-related pathological conditions (Figure 3). Thus, chronic stimulation of mitochondrial turnover by enhancing mitophagy is a promising approach to delay age-related diseases and to extend health- and lifespan.

## Author Contributions

OK, GC, and GK wrote the manuscript and generated the figures.

## Conflict of Interest

OK and GK were scientific co-founders of Samsara Therapeutics. The remaining author declares that the research was conducted in the absence of any commercial or financial relationships that could be construed as a potential conflict of interest. The reviewer MD declared a past co-authorship with one of the authors GK.
